# Allelic variation at the *rpv1* locus controls partial resistance to *Plum pox virus* infection in *Arabidopsis thaliana*

**DOI:** 10.1186/s12870-015-0559-5

**Published:** 2015-06-25

**Authors:** S. Poque, G. Pagny, L. Ouibrahim, A. Chague, J-P Eyquard, M. Caballero, T. Candresse, C. Caranta, S. Mariette, V. Decroocq

**Affiliations:** INRA, UMR 1332 Biologie du Fruit et Pathologie, F-33140 Villenave d’Ornon, cedex France; Université de Bordeaux, UMR 1332 Biologie du Fruit et Pathologie, F-33140 Villenave d’Ornon, cedex France; INRA-UR1052, Genetics and Breeding of Fruits and Vegetables, Dom. St Maurice, CS 60094, F-84143 Montfavet cedex, France; Current address: INRA, UMR 1202 Biogeco, F- 33610 Cestas, France; Current address: Univ. Bordeaux, UMR1202 Biogeco, F-33400 Talence, France; Current address: Department of Plant Pathology, National Chung Hsing University, Taichung, 402 Taiwan

**Keywords:** Partial resistance, recessive resistance, QTL mapping, association mapping, PPV, *Plum pox virus*, *Arabidopsis* thaliana, cPGK

## Abstract

**Background:**

Sharka is caused by *Plum pox virus* (PPV) in stone fruit trees. In orchards, the virus is transmitted by aphids and by grafting. In *Arabidopsis*, PPV is transferred by mechanical inoculation, by biolistics and by agroinoculation with infectious cDNA clones. Partial resistance to PPV has been observed in the Cvi-1 and Col-0 *Arabidopsis* accessions and is characterized by a tendency to escape systemic infection. Indeed, only one third of the plants are infected following inoculation, in comparison with the susceptible L*er* accession.

**Results:**

Genetic analysis showed this partial resistance to be monogenic or digenic depending on the allelic configuration and recessive. It is detected when inoculating mechanically but is overcome when using biolistic or agroinoculation. A genome-wide association analysis was performed using multiparental lines and 147 *Arabidopsis* accessions. It identified a major genomic region, *rpv1*. Fine mapping led to the positioning of *rpv1* to a 200 kb interval on the long arm of chromosome 1. A candidate gene approach identified the chloroplast phosphoglycerate kinase (*cPGK2*) as a potential gene underlying the resistance. A virus-induced gene silencing strategy was used to knock-down *cPGK2* expression, resulting in drastically reduced PPV accumulation.

**Conclusion:**

These results indicate that *rpv1* resistance to PPV carried by the Cvi-1 and Col-0 accessions is linked to allelic variations at the *Arabidopsis cPGK2* locus, leading to incomplete, compatible interaction with the virus.

**Electronic supplementary material:**

The online version of this article (doi:10.1186/s12870-015-0559-5) contains supplementary material, which is available to authorized users.

## Background

Potyviruses represent about 20 % of known plant viruses and are economically among the most important threat for vegetable and fruit trees crop species. Among them, *Plum pox virus* (PPV) infects *Prunus* species (stone fruits) and causes sharka disease which devastates fruit and plant production, significantly impacting crop quality and yield. Over the last 30 years, Sharka costs to the industry worldwide have been estimated at 10 billion Euros [1]. Unfortunately, only a few sources of natural resistance are available in *Prunus* hosts. In order to expand the range and understand the nature of resistance sources, we are investigating new resistances to PPV in the model host plant *Arabidopsis thaliana*.

*Arabidopsis* is commonly used for the acquisition of knowledge on basic plant biology and on adaptation to biotic or abiotic stress. Its small size, rapid life cycle and small genome size of ~150 Mb make it an ideal model plant for biotechnological and genetical characterization of plant disease resistance. Moreover *Arabidopsis* is susceptible to various viral pathogens such as potyviruses (e.g. *Turnip mosaic virus*; *Tobacco etch virus*; *Lettuce mosaic virus* (LMV) or PPV), cucumoviruses (*Cucumber mosaic virus*), luteoviruses (*Beet western yellow virus*) and others, making it an ideal host for the identification of genes underlying susceptibility or resistance to viral infection [2, 3].

Indeed, three distinct mechanisms of resistance to the PPV and LMV potyviruses have recently been identified in *Arabidopsis* [4–6], two of which show a recessive genetic determinism. Viruses are obligatory intracellular parasites highjacking the host cell machinery to complete the different steps of their infectious cycle. The disruption of compatible interactions between host and viral factors during replication or translation (or any other viral function) of the viral genome may lead to the failure of the corresponding infection step, operationally resulting in a recessive resistance [7]. This kind of resistance seems to be more frequent for plant/potyvirus pathosystems, representing 40 % of the resistances identified, up to now, in the natural diversity of crop species. It is worth noting that most of the studies on recessive resistance to potyviruses published to date identified genes encoding the translation initiation factors eIF4E and eIF4G or their isoforms [8].

Recessive resistances against PPV and another potyvirus, *Watermelon mosaic virus* (WMV), were identified in the ‘Cape Verde Island’ *Arabidopsis* ecotype (Cvi-1 and Cvi-0, respectively) following mechanical inoculation. These resistances were mapped to the same interval on chromosome 1 [6, 9] and the corresponding genes were respectively named *rpv1* and *rwm1*. In the case of *rwm1*, a chloroplast phosphoglycerate kinase (cPGK2) has recently been identified as responsible for WMV resistance [9]. This cytosolic isozyme of chloroplastic PGK is a ubiquitous monomeric enzyme that can also play roles in DNA repair [10] or, in the case of the paramyxovirus *Sendai virus*, in stimulation of mRNA transcription during the elongation step [11]. In plants, it was shown that lowering cPGK levels reduced the accumulation of *Bamboo mosaic virus* (BaMV), a member of *Potexvirus* genus [12].

Another recessive resistance against PPV has been identified among *Arabidopsis* accessions of diverse origins following *Agrobacterium*-mediated inoculation [5]. It was designated *sha3* for “sharka resistance” and appears to be unlinked to *rpv1* as it maps at the bottom of linkage group 3. Variation at the *sha3* locus restricts PPV long distance movement and viral systemic infection [5]. In the present study, genetic analysis and linkage mapping of recombinant inbred line (RIL) populations and genome wide association mapping in a multiparental population were used to demonstrate the existence of *rpv1* resistance alleles in both Cvi-1 and Col-0 and to identify *cPGK2* as the cellular gene underlying this resistance to PPV in *Arabidopsis*. We also confirm that the *rpv1*-driven tendency to escape PPV infection is distinct from the *sha3* resistance mechanism and that it is specific to the method of inoculation.

## Results

### Testing different inoculation methods on the Cvi-1, Col-0 and L*er Arabidopsis* accessions

**Fig. 1:**
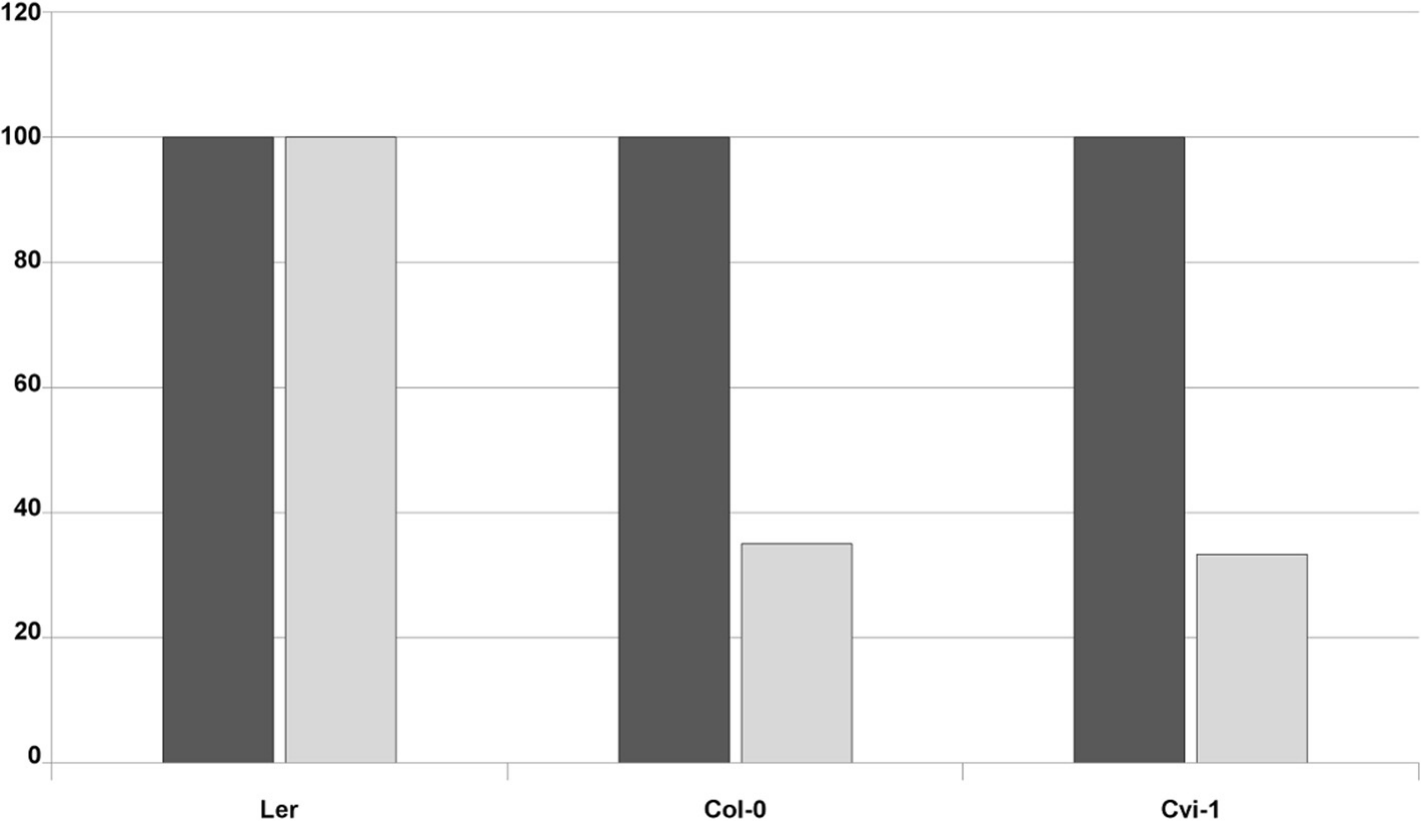
Percentage of infected plants among susceptible (L*er*) and partially resistant (Col-0, Cvi-1) accessions following agroinoculation (dark bars) or mechanical inoculation (light bars). The results presented are those of representative experiments involving 12 to 24 *Arabidopsis* plants per condition. The infection status was determined by an ELISA assays performed on non-inoculated tissues 21 days post inoculation

*Arabidopsis thaliana* can be experimentally inoculated with *Plum pox virus* (PPV) using different methods: i) mechanically [6, 13], ii) by biolistics [14] and iii) by agroinoculation [5]. In the first case, the virus is delivered as an encapsidated virion while purified DNA molecules are transferred by *Agrobacterium* or shooting. To test the effect of the inoculation method (or of the viral form) on the outcome of the *Arabidopsis*/PPV interactions, several accessions were inoculated in parallel with the three methods described in the Material and methods’ section. The accumulation of PPV-R in the L*er*, Cvi-1 and Col-0 accessions was estimated at 21 days post inoculation (dpi) by ELISA. Surprisingly, while the three accessions are fully susceptible to PPV infection after biolistic (not shown) or agroinoculation, both Cvi-1 and Col-0 showed a constant tendency to escape systemic infection when inoculated mechanically (Figure 1). Indeed, viral accumulation was detected in only 33 to 35 % of inoculated plants. According to Lecocq et al. [15], partial resistance is, in some cases, based on the tendency to escape infection and may be characterized as a lower probability of infection than that of susceptible plants, using the same level of inoculum, which is the case for Cvi-1 and Col-0 when mechanically infected by PPV. This observed phenotype in response to PPV infection will thus be named “partial resistance”, hereafter.

We also checked if the phenotype observed was linked to a true, partial resistance mechanism or if the infected plants carried a PPV variant that had evolved the ability to overcome the Col-0 resistance. We carried out serial passage of PPV into Col-0 as described in the material and methods section. In the two replicate experiments, PPV infection rate in the back inoculated Col-0 plants reached only 33.3 to 37.5 %, demonstrating the stability of the resistance phenotype. It thus appears that the partial block in PPV systemic infection in *Arabidopsis* dependent on the inoculation method is stable, and that this partial resistance in Cvi-1 and Col-0 is observed only when plants are mechanically inoculated. This resistance is overcome upon biolistic or *Agrobacterium*-mediated inoculation. Cvi-1 had previously been shown to be resistant to PPV upon mechanical inoculation and the locus involved, *rpv1*, mapped to a chromosome 1 interval [6].

### Genome Wide Association mapping of *Arabidopsis* resistance after mechanical inoculation

We recently showed using a larger set of *Arabidopsis* accessions that resistance to PPV inoculated by *Agrobacterium* is controlled by a different locus, named *sha* 3 gene [5]. In an effort to evaluate that the *rpv1* resistance is unrelated to the *sha3* PPV resistance locus, a set of 147 *Arabidopsis* accessions (see Additional file 1: Table S1) previously used to identify *sha3* was mechanically inoculated with PPV. The experiment was duplicated and the broad-sense heritability, calculated as described in material and methods, of PPV resistance reached 0.82 and 0.83, respectively.

Fisher’s exact test identified SNPs significantly linked to resistance to PPV. Notably, 15 of the 500 SNPs (Additional file 2: Table S2A to S2D) with the lowest *p*-values were located on chromosome one. This region coincides with a previously identified locus associated to resistance to PPV mechanical inoculation in the Cvi-1 accession and named *rpv1* [6]. When using the quantitative data (normalized optical density values, see material and methods), 43, 31 and 31 SNPs, out of the 500 SNPs with the lowest *p*-values, belong to the same *rpv1* genomic region, with the Wilcoxon, PLINK and EMMA methods, respectively (Additional file 2: Table S2B, C and D). In addition, SNPs localized to the *sha3* interval were also detected among the 500 most significant SNPs, thereby confirming that both *rpv1*- and *sha3*-driven resistance mechanisms are present, concomitantly, in the population of natural *Arabidopsis* accessions. However, since we cannot rule out that spurious, false positive association may arise from population structure, a traditional linkage mapping in recombinant inbred line populations was conducted, in order to confirm and fine map *rpv1*.

### Linkage mapping of resistance to PPV systemic accumulation after mechanical inoculation of an *Arabidopsis* multiparental recombinant population

**Fig. 2:**
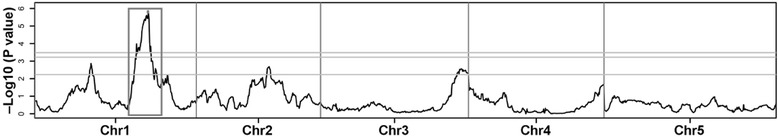
Genome wide association mapping (GWAM) of the resistance to PPV in the MAGIC population. The y axis represent –Log10(P value) obtained for each SNP throughout the five *Arabidopsis* chromosomes. Chr: chromosome. lsmean of quantitative data were analysed as described in [5, 16]. The threshold *P* value (dotted line) was calculated by Bonferroni

Four hundred and thirty-five of the 527 MAGIC (Multiparent Advanced Generation Inter-Cross) recombinant inbred lines described by Kover et al. [16] were evaluated in a three random blocks design following a mechanical inoculation. A significant block effect was detected and a QTL mapping analysis was performed using data from each block separately, as well as using a lsmeans model, accounting for the effect of each block. The broad-sense heritability of PPV resistance for the MAGIC lines was calculated from the variance analysis (see material and methods) and reached 0.77. Interestingly, the same genomic region was identified when using data from block one and three separately or from the mean values of the three blocks. Analysis of the variation in susceptibility to PPV infection for the first and third block identified one QTL on linkage group 1 at position 19,778,790 bp (−log10 (*p-*value) = 4.07) and 22,286,231 bp (−log10 (*p-*value) = 3.62), respectively. By using lsmeans values, a major QTL was identified in the same genomic region as for blocks 1 and 3, with a maxima of the -log10 (*p-*value) of 5.80 (Table 1A and Fig. 2). This region coincides with *rpv1*. Surprisingly, even if point wise *p-*values were significant at the 1 % level, this analysis failed to identify the *sha3* PPV resistance locus which had previously been identified in the same MAGIC RILs population using *Agrobacterium*-mediated PPV inoculation [5]. From the QTL analysis of the MAGIC lines, the genome of each line was reconstructed as a mosaic of the founder haplotypes [16]. Based on this reconstruction, it was possible to determine that the three founders contributing the QTL detected in the MAGIC lines were Col-0, Can-0 and Ws. In comparison, PPV agro-inoculation of the same MAGIC lines had shown that the *sha3* resistance was contributed by two unrelated founders, Hi-0 and Sf-2 [5].

**Table 1:** Identification of Arabidopsis genomic regions controlling restriction of PPV infection in bi- and multi-parental populations

A)											
Multiparental progeny	Type of population/Nb of RILs		Set of markers used for the analysis †		Linkage group	Peak in Bp	Peak SNP	Interval in Bp	LogP	Genome-wide P-Value	
MAGIC	RIL/435^1^		1,260 SNPs		LG1	21,665,899	MN1_21669564	19,515,673 - 22,286,231	5.80	0.002	
B)											
Bi- parental progenies	Type of population/Nb of RILs	Nb Markers †	Parental phenotypes		Predicted locus location						
			Parents 1	Parents 2	linkage group	Resistant parental allele	Marker interval	Interval in bp	Maximum LOD (IM) ‡*	P-Value (Cavatorta et al.)‡*	R^2^
JEAxCol-0	RIL F8/188^1^	87	S	R	LG1	Col-0	c1_19478/c1_23381	19,477,618 – 23,381,469	15.99	65.48	33.10%
JEAxCol-0	RIL F8/120^1^	87	S	R	LG1	Col-0	c1_19478/c1_23381	19,477,618 – 23,381,469	12.5	45.431	38.90%
JEAxCol-0	RIL F8/250^1^	97	S	R	LG1	Col-0	F6D8-SSLP1/RCVI-32	19,624,624 - 22,181,333	21.78	81.865	34%

^1^ In four repeats for the JEA x Col-0 RILs population and triplicates for the MAGIC lines. † Number of markers used to build the core genetic map (SSR or SNP) [32]. ‡ Detected by Interval Mapping -IM- or Krustal Wallis -KW-. * Significative after 1,000 permutations and at 95 % statistical confidence. LogP is equivalent to the -log10(*p-*value). bp: base pairs, Maximum LOD: score associated with the peak of the LOD plot using Map QTL, R^2^: Proportion of the phenotypic variation explained by the peak of the LOD plot using multiple QTL mapping (explained variance)

These results therefore suggest that different resistance genes may be uncovered when using different inoculation methods. The differences observed using the two inoculation methods could be due to either 1) a larger genetic effect of *rpv1* over *sha3* that would hide the relative effect of the second mechanism when using mechanical inoculation, 2) a loss of the *sha3*-driven resistance when using mechanical inoculation or, alternatively, a loss of the *rpv1*-driven resistance upon agroinoculation, or 3) a difference in timing, i.e. *rpv1*-driven resistance taking place earlier in the viral life cycle than the *sha3*-driven mechanism. In order to test the first hypothesis, the analysis was repeated removing the MAGIC lines that possess a higher probability of having the genotype of the resistant founders in the *rpv1* region. The recalculated point wise *p-*value at the *sha3* locus was decreased, suggesting that removing partially the effect of the *rpv1* locus improved the detection of the *sha3* locus (data not shown). Therefore, even if the *sha3* QTL was difficult to detect when plants were mechanically inoculated, resistance to PPV systemic infection in the MAGIC lines appears to be controlled by at least two different loci, *rpv1* and *sha3,* respectively.

### Linkage mapping of the *rpv1*-driven resistance trait in the recombinant JEAxCol-0 population

In order to confirm the occurrence of *rpv1* in the Col-0 background, two distinct sets of biparental JEA × Col-0 recombinant inbred line population were mechanically challenged with PPV, in a completely randomized design in four independent blocks. These sets were constituted of 188 and 120 individuals, respectively. The experiment on the set of 188 RILs was repeated twice over two years.

**Fig. 3:**
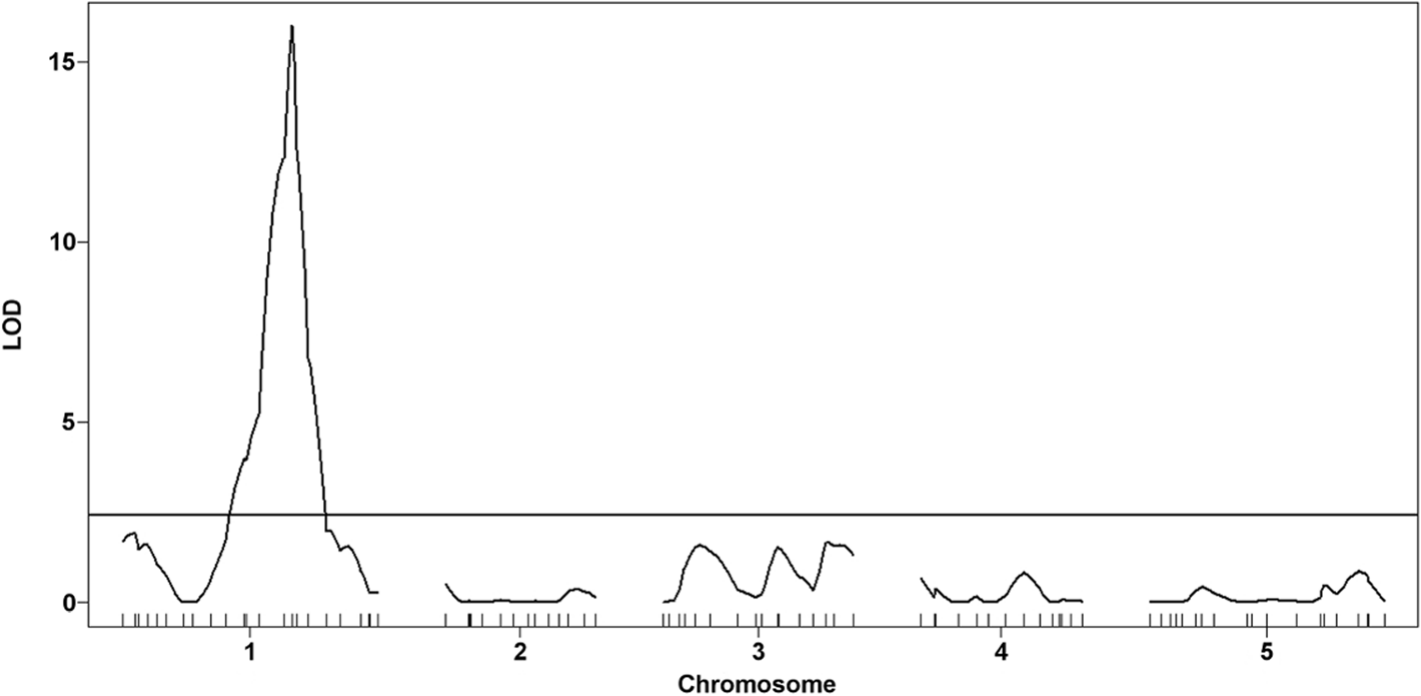
Linkage mapping of the recessive resistance to PPV in an F8 JEAxCol-0 RIL population. The y axis represents the LOD score obtained by interval mapping (IM) on the first set of 188 RILs

Results show that variances are not significantly heterogeneous in all experiments (Levene's test *p-*value for both sets of 188 RILs were 0.38 and 0.33, respectively), and results of an ANOVA test confirmed that the block design had no significant effect on viral infection (*p*-value for both sets of 188 RILs were 0.3902 and 0.1898, respectively). As a consequence, the QTL analysis was performed on pooled blocks results, using the MapQTL and RQtl softwares. A Kruskal-Wallis test was first performed, to detect markers linked to the resistance to PPV. An approximate LOD score was then computed through interval mapping. Table 1B summarizes Krustal -Wallis *p*-values and LOD scores for each QTL. In the 188 RILs experiment, the effect of only one locus was observed, with a LOD score of 15.99 and a R^2^ of 33.1 %. This single locus is located on chromosome one, between 60.5 cM to 69.1 cM (19,477,618 bp to 23,381,469 bp) (Fig. 3), the same region previously identified in Cvi-1 [6] and in the MAGIC lines. The second set of JEA x Col-0 recombinant lines used was composed of 120 RILs selected so that they display at least one recombination event over the 60.5-69.1 cM interval identified above. Phenotyping of the set of 120 RILs resulted in the mapping of one single locus co-localizing with *rpv1* (Table 1B) but with a higher effect (R^2^ up to 38.90 %).

It appears here that Cvi-1 and Col-0 are sharing the same genomic region. A JEA × Col-0 F1 population was tested for resistance to PPV mechanical inoculation and a majority of the plants (75 %) resulted positive. The segregation ratio in F2 Cvi-1 × L*er* progenies [6] as well as the fact that the JEA × Col-0 F1 population is susceptible indicate that both populations display a recessive resistance to PPV. However, since the interval is still rather large, we cannot rule out, at this stage, an overlapping of two distinct loci. We thus performed the fine mapping of this region which controls resistance to PPV mechanical inoculation and allelism tests.

### Fine-mapping of the *rpv1* locus in near isogenic backgrounds

**Fig. 4:**
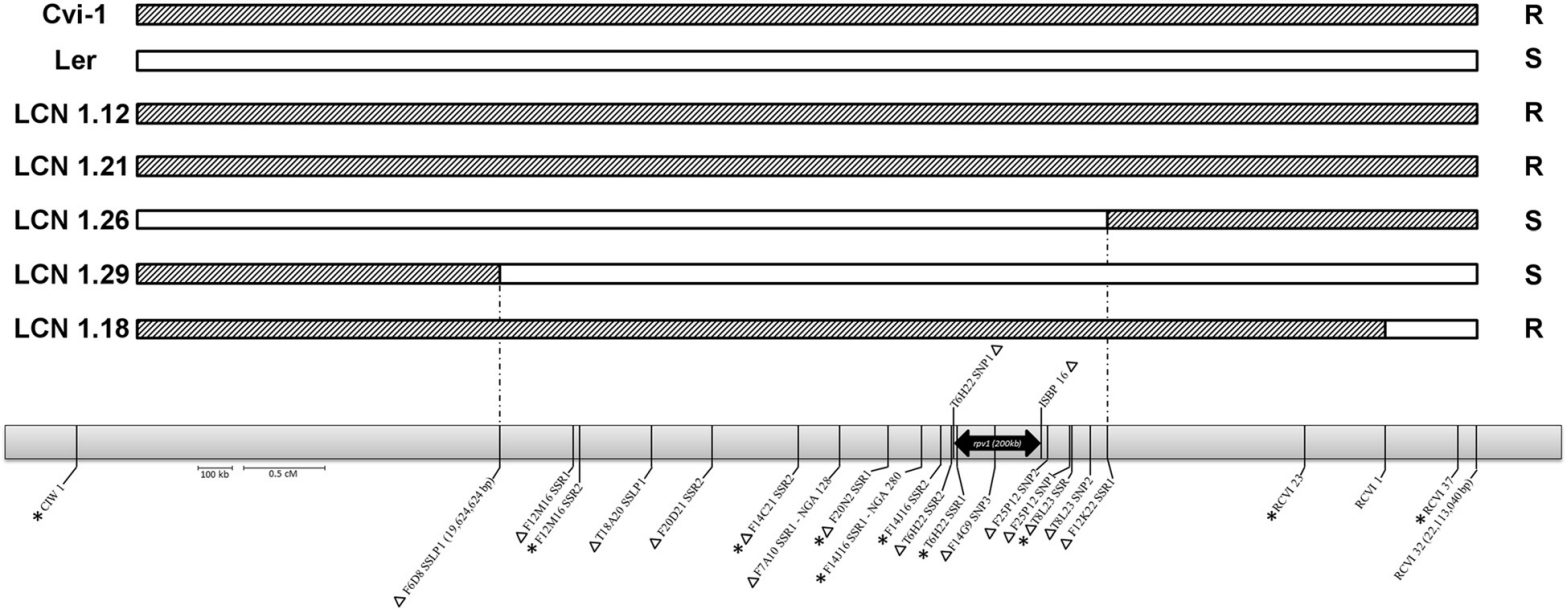
Schematic representation of near-isogenic lines (NILs) and markers used to fine map *rpv1.* ^Δ^ Markers used to fine map *rpv1* in Near-isogenic lines; *Markers used to improved linkage mapping in JEA × Col-0 recombinant inbred lines; R for resistant accession (Cvi-1) or near-isogenic lines (LCN); S for susceptible accession (L*er*) or near-isogenic lines

To avoid any epistatic interactions with other loci, the fine mapping of *rpv1* was performed in near isogenic lines (NILs) originating from a Cvi-1 × L*er* recombinant inbred line population [17]. The procedure was conducted in three steps as described in the material and methods section. The first step allowed us to determine the upper and lower borders of the *rpv1* locus in Cvi-1 (Fig. 4), as depicted in the NILs LCN1.29 and 1.26. The identified recombination points were flanked by two markers, F6D8 SSLP1 and F12K22 SSR1 (Additional file 3: Table S3), and delineated an interval of 1.8 Mb (Fig. 4).

The second fine-mapping step allowed to reduce the *rpv1* interval down to 460 kb, between markers T6H22 SSR2 and F12K22 SSR1. The third step consisted in a second run of fine-mapping, this time using a LCN1.26 (susceptible) × LCN1.21 (resistant) F2 population of 840 individuals. In this case, all lines were screened with the T6H22 SSR2, ISBP 16, T8L23 SSR and F12K22 SSR1 markers. This allowed narrowing down the *rpv1* interval to 260 kb, between positions 20,971,975 and 21,232,895 on the long arm of chromosome 1 (Fig. 4).

### The Col-0 and Cvi-1 resistances involve the same gene

In order to see if the Col-0 and Cvi-1 resistances involve the same gene, we performed an allelism test. Col-0 was crossed with a resistant near isogenic line carrying the *rpv1* genomic region of Cvi-1, namely LCN1.18 (See Fig. 4). The corresponding F1 progeny was mechanically challenged with PPV and 100 % of tested plants were observed to be resistant. Since partial resistance in Col-0 and Cvi-1 is recessive, this allelism test demonstrates that *rpv1* is allelic in both accessions.

### Further characterization of the breakdown of the *rpv1-*mediated resistance upon biolistics or agroinoculation

**Fig. 5:**
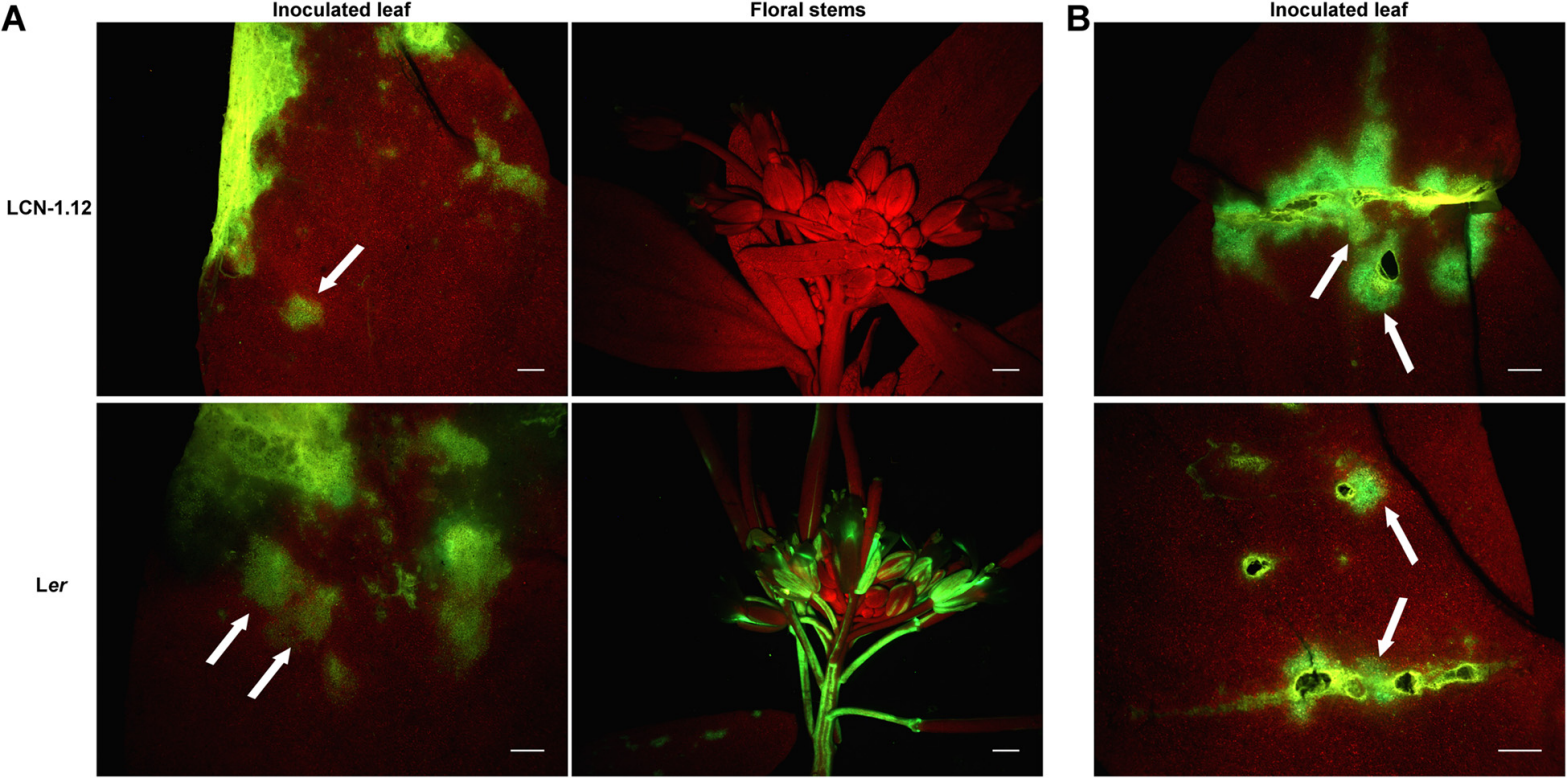
Green fluorescence protein (GFP)-tagged Plum pox virus (PPV-R) behavior into inoculated leaves of Ler and a PPV-resistant LCN line (LCN-1.12). Photographs under a UV stereomicroscope of GFP accumulation in L*er* and LCN 1.12, inoculated (first column) and systemic tissues (second column) after mechanical inoculation with pICPPVnkGFP (**a**) and inoculated leaf after agro-inoculation with pBINPPVnkGFP (**b**). White arrows point out inoculation area

In order to further characterize the *rpv1*-mediated resistance and to ensure that it had been properly mapped, L*er*, Cvi-1 and ten selected LCN NILs (five PPV-resistant and five PPV-susceptible LCN near-isogenic lines) were inoculated in parallel using three techniques: mechanical inoculation, agro-inoculation or biolistics. In each case, the same viral inoculum, derived from PPV-R, was used. Mechanical inoculation of the Cvi-1, L*er* and near-isogenic LCN lines provided viral infection ratios similar to those shown in Fig. 1. In comparison, when the five PPV-resistant LCN lines (LCN 1.12, 1.18, 1.21, 1.22, 1.23) were inoculated either by agroinoculation or by biolistics, 100 % of the plants showed PPV systemic accumulation (not shown). Fluorescence microscopy observation of mechanically inoculated leaves of L*er* and of the PPV-resistant LCN lines (see LCN1.12 as a representative example in Fig. 5A) revealed clear PPV accumulation, demonstrating that *rpv1* does not prevent multiplication in inoculated leaves but only affects PPV systemic infection of non-inoculated tissues. However, an effect of *rpv1* on a reduction in the accumulation rate in inoculated leaves could not be ruled out.

### cPGK2: a potential candidate for *rpv1*

**Fig. 6:**
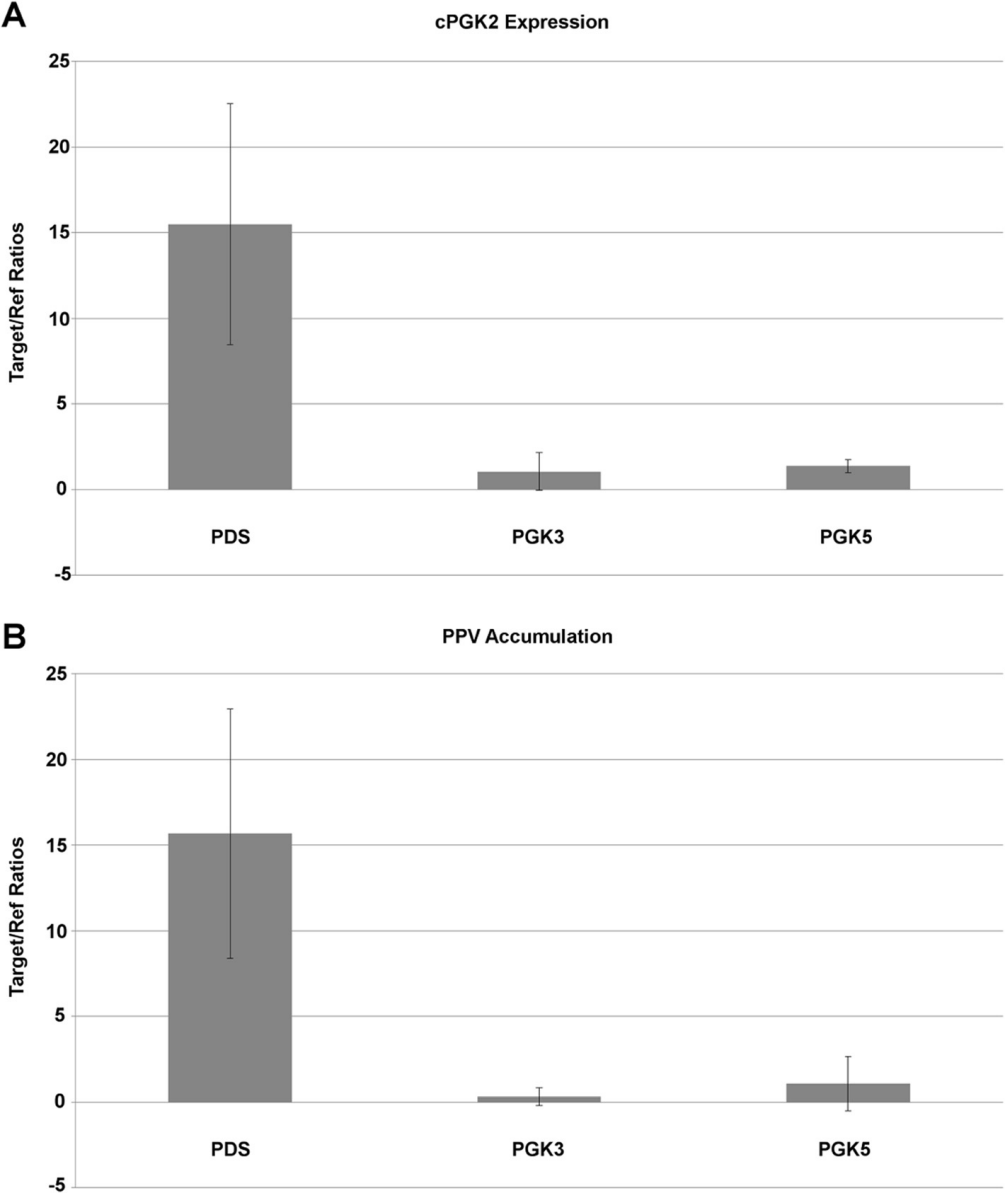
Effect of the viral-induced silencing of the *cPGK2* gene on PPV accumulation in *Nicotiana benthamiana*. **a**,The accumulation levels of *cPGK2* transcripts and **b**, PPV RNA were measured by quantitative RT-PCR in the non-inoculated leaves at 6 dpi. The values represent means (± sd) of fold changes relative to the control (Mock). Each sample includes four to six biological replicates. The RNA levels were normalized to that of Nb*EF1*. Means and standard errors are displayed as vertical bars. The phytoene desaturase (PDS) was used as positive control

Studies with the *Watermelon mosaic virus* (WMV) – *Arabidopsis thaliana* pathosystem have identified in Cvi-1 a recessive resistance gene (*rwm1*) that maps to the same region as the *rpv1* locus. Using a combination of fine mapping and functional validation, *rwm1* has recently been shown to correspond to a gene coding for a chloroplast phosphoglycerate kinase (cPGK2) [9]. Given the co-localization of the *rwm1* and *rpv1* resistances, the possibility that the same *cPGK2* might contribute to the resistance to PPV systemic accumulation analyzed here was evaluated. Similarly to Ouibrahim et al. [9], a TRV-based VIGS system was used to knock-down *cPGK2* expression in *Nicotiana benthamiana* and to evaluate its impact on PPV accumulation. The entire experiment was repeated twice and means results between these two experiments are presented in Fig. 6. The results obtained show that the levels of the chloroplast PGK2 mRNA in the PGK-5 and PGK-3-inoculated plants were reduced by about 90 % as compared to control plants inoculated with the PDS construct. In the same plants, PPV accumulation in systemic, non-inoculated leaves was reduced by over 90 % in PGK-3- and PGK-5-silenced plants. Taken together, these results suggest that the chloroplast PGK2 is required for efficient PPV accumulation in *N. benthamiana*.

### *cPGK2* is down-expressed in *Arabidopsis* Col-0 rosette leaves but not in Cvi-1.

While both Cvi-0 and Cvi-1 *cPGK2* genes (At1g56190) display a non-synonymous mutation [9], no allelic difference was identified in Col-0, in comparison to L*er*. We thus hypothesized that resistance to PPV in Col-0 is linked to a transcriptional regulation of the *cPGK2* gene. Total RNA was extracted from Col-0, L*er* and Cvi-1 rosette leaves at inoculation time and *cPGK2* expression was tested by quantitative Real Time Reverse Transcription PCR (Q-RT-PCR) analysis. The expression level of *cPGK2* was compared to the At2g36060 *Arabidopsis* reference gene [18] (Additional file 4: Fig. S1). Interestingly, *cPGK2* in Col-0 rosette leaves is two-fold downregulated in comparison with L*er* and up to 14 times less expressed in comparison with Cvi-1.

## Discussion

In the present study, we report the identification in *Arabidopsis* of a genomic region associated with partial resistance to PPV systemic accumulation upon mechanical inoculation. In order to fine map this region a combination of linkage mapping in RIL and NIL populations and of genome wide association mapping were used. Each of the bi- and multi-parental linkage mapping experiments detected a major and recurrent locus that had previously been mapped by Sicard et al. [6] and named *rpv1*. Allele(s) which determine this resistance trait are present in both the Cvi-1 and Col-0 accessions, as well as in Can-0 and Ws as shown in the MAGIC experiment. An allelism test indicated that restriction of PPV infection in the Cvi-1 and Col-0 accessions is controlled by the same gene. We also showed that *rpv1* is distinct from the previously identified *sha3* locus.

Indeed, in a recently published report, the resistance to PPV systemic accumulation following *Agrobacterium*-mediated inoculation has been analyzed in the same set of multiparental recombinant lines, using the same viral isolate (PPV-R) [5]. This work identified loci controlling PPV systemic accumulation (in particular the *sha3* locus) but not the *rpv1* locus. This could be linked to the finding that the *rpv1*-controlled recessive resistance is overcome when PPV is inoculated using either agro-inoculation or biolistics. This phenomenon can be explained either by an over-load of PPV inoculum when using the more efficient agroinoculation or biolistics techniques, or by differences in the biological form of the virus when delivered: an encapsidated virion for mechanical inoculation, purified infectious cDNA molecules in the other techniques. Alternatively, the use of a more effective inoculation method may result in a higher number of initially infected cells or in a higher viral load in those cells, allowing the virus to overcome the *rpv1* resistance mechanism. Such a scenario has been observed previously for both *Cauliflower mosaic virus* [19] and *Plantago asiatica mosaic virus* [20] but in both cases, the resistance involved was dominant. This result also poses the question of the interest and of the durability of the *rpv1*-driven resistance mechanism if transferred to *Prunus* hosts.

A recent study in WMV – *Arabidopsis* pathosystem identified *rwm1*, a recessive resistance gene in Cvi-0 which co-localizes in the *rpv1* interval determined here. In Cvi-0, *rwm1* determines recessive resistance to WMV, with incomplete penetrance depending on the WMV isolate (up to 16 % of plants are infected when infected by WMV-LL2 or –AUST89) [9]. This incomplete penetrance is speculated to be an environmental effect, as it is in particular affected by light exposure during inoculation of the plants. In the case of *rpv1*, the incomplete penetrance can attain a level of 33-35 % of the plants. In the experiments reported here, the different populations were tested at the same period of the year and in the same environmental conditions (e.g. same greenhouse, same time of the day for inoculations). In order to differentiate selection of resistance-breaking viral isolate from true, partial resistance, populations of PPV were allowed to evolve for 10 consecutive 21-day serial passages in the Col-0 accession. By the end of this experiment, PPV did not show any increased virulence. Thus, *rpv1* can be considered as a locus controlling partial resistance to PPV infection. The term “partial resistance” and its various interpretations are widely used in plant/pathogen genetics, in particular in describing plant/virus interactions. In most cases it has been associated with quantitative resistance and described as a reduction of disease intensity, or of pathogen accumulation, rather than the absence of disease [21, 22]. It can be attributed to a lower viral multiplication or accumulation, with the virus able to infect the host plant systemically but remaining at a lower concentration in plant tissues [23, 24]. Some authors also used the term partial resistance when viruses are restricted to specific tissues or to specific stages of the host plant development [25–27]. Finally, others describe partial resistance as an absence of symptoms despite a normal viral accumulation in systemic tissues (tolerance).

In the case of the *rpv1* resistance, partial resistance to PPV infection in Cvi-1 and Col-0 is characterized by the tendency to escape systemic infection upon mechanical inoculation. Given the recessive nature of the resistance, this could be explained by a weaker interaction between host factor(s) and viral proteins. Among potential candidate genes present in the restricted *rwm1* interval, Ouibrahim et al. [9] discovered a non-synonymous mutation (S78G) in the Cvi-0 and Cvi-1 *cPGK2*. They demonstrated, using a TRV-based virus induced gene silencing system in *N. benthamiana,* that WMV accumulation was affected by the reduction of the AT1G56190 *cPGK2* transcripts. The same approach was used here to demonstrate that *cPGK2* expression is also necessary for efficient PPV accumulation in *N. benthamiana* systemic leaves. Therefore, a likely hypothesis is that *RPV1* might be a functional *cPGK2* gene, in PPV susceptible accessions such as L*er*. Partial resistance in the Cvi-1 accession can be potentially explained by a weaker interaction between Cvi-cPGK2 and PPV protein(s) as a result of the identified S78G mutation. However, the Col-0 *cPGK2* gene does not display nucleotide variation in comparison with L*er* and *rpv1* resistance in Col-0 might occur at another level, possibly *cPGK2* reduced expression. Since *rpv1* is controlling a recessive resistance mechanism, susceptibility to the virus is thus dependent on the amount of cPGK2 proteins available for full compatible interaction.

In this respect, it is worth noting that in the complete *Arabidopsis* transcriptome data base (CATdb) the L*er cPGK2* transcript level was not significantly different between PPV-infected and mock inoculated plants (http://urgv.evry.inra.fr/cgi-bin/projects/CATdb/consult_project.pl?project_id=118) while Babu et al. [28] showed an induction of *cPGK2* expression in Col-0 leaf tissues 17 days post PPV inoculation. In our case, we showed a significantly lower expression of *cPGK2* in Col-0 in comparison with the susceptible L*er* accession. In consequence, a limitation of *cPGK2* transcripts in the inoculated leaves could explain the partial resistance of Col-0 to PPV infection. However, while our results are in agreement with experiments conducted with WMV [9], we showed here that *cPGK2* gene silencing affects viral accumulation in *N. benthamiana*, not the number of plants truly infected.

## Conclusion

Most of the studies on recessive resistance published to date describe alleles of genes encoding the translation initiation factors [8] as mediators of virus resistance. The present report describes a PPV recessive resistance mechanism potentially involving a chloroplast phosphoglycerate kinase. The identification of a new PPV resistance mechanism, distinct of the translation initiation complex, is important for developing novel strategies for resistance gene pyramiding in stone fruit crop species. However, deployment of such resistance specific to the inoculation method has to be considered carefully and should be combined with other, more general, resistance mechanisms.

## Methods

### Plant material

We used two *Arabidopsis* populations of recombinant inbred lines (RILs), one derived from a cross between Col-0 (Columbia) (186AV in the VNAT collection, N1092 in the NASC collection) and JEA (25AV), and the other from a cross between Cape Verde Islands (Cvi-1) and Col-0. Both of them were developed by VNAT INRA of Versailles (http://publiclines.versailles.inra.fr/).

For fine-mapping, we also used the so-called LCN near isogenics lines [17]. They originated from a Landsberg *erecta* (L*er*) × Cvi-1 cross in which Cvi-1 genomic regions were introgressed into a L*er* background. From the LCN NILs, we generated two F2 populations as follows: one by backcrossing the PPV resistant LCN 1.12 in L*er* and the second by crossing the LCN1-26 (susceptible) with LCN 1.21 (resistant). F1 heterozygous plants were checked before selfing with the AthGENEA marker for the first LCN1.12 × L*er* cross and with T18A20 SSLP1 for LCN1.26 × LCN1.21 (see markers listed in Additional file 3: Table S3).

*Arabidopsis* natural accessions for genome-wide association study and the MAGIC (Multiparent Advanced Generation Inter-Cross) recombinant population were obtained from the Nottingham *Arabidopsis* Stock Centre (NASC) (http://szlapncs01.nottingham.ac.uk/).

Plants were grown in a BL-3 containment greenhouse under temperature and humidity controlled conditions (20 °C and relative humidity of 60 %).

### Viral material

*Arabidopsis thaliana* is inoculated with PPV using different methods: i) mechanically using an inoculum derived from pICPPVnkGFP-infected *Nicotiana benthamiana* leaves [6, 13], ii) by biolistics using a pICPPVnkGFP cDNA clone [14] and iii) by agroinoculation with a pBINPPVnkGFP clone introduced in *Agrobacterium tumefaciens* [5]. In each case, the two viral clones, *i.e.* pICPPVnkGFP and pBINPPVnkGFP, are derived from the same PPV-R isolate, which belongs to the PPV-Dideron strain [29]. Construction of pBINPPVnkGFP containing the full-length nucleotide sequence of PPV-R coupled with the green fluorescence (GFP) protein has been described by Fernández-Fernández et al. [30].The serial passage assay was done by using homogenates from PPV-positive Col-0 plants at 21 dpi to inoculate 24 to 48 Col-0 in 10 successive passaging assays. The experiment was repeated twice, in parallel.

### PPV resistance phenotyping

The pICPPVnkGFP virus clone was mechanically inoculated on the rosette leaves at four weeks after sowing. Virus infection was scored at 21 days post inoculation (dpi) in non-inoculated tissues (flower stems or newly developed rosette leaves). The inoculum was derived from pICPPVnkGFP-infected *Nicotiana benthamiana* leaves. At 21 dpi, viral accumulation was estimated for each individual plant using double antibody sandwich (DAS) ELISA assays [6]. Optical densities (OD) were normalized using the PPVnkGFP infected *Nicotiana benthamiana* positive control deposited on every ELISA plate of an assay. Quantitative data were normalized relative to the value of the PPVnkGFP infected *N. benthamiana*, which was set at 100. In the case of RILs, the final viral accumulation value is the average of normalized measurements from all PPV-inoculated replicates of each RIL.

### Mapping of the genetic determinants in bi-parental populations

The F8 JEAxCol-0 RIL population (28RV, http://publiclines.versailles.inra.fr/rils/index) is comprised of 455 lines genotyped with 87 markers [31]. Two sets of 188 and 120 individuals, both parents, and the PPV resistant E6 eIFiso4E loss-of-function mutant [13], that served as a negative control, were challenged with PPV. Experiments were set up in a 4-blocks random design and the 188 set was duplicated over two years during the same winter period, while the next 120 RILs, recombinant over the candidate *rpv1* region, were tested only once. For both data sets (188 and 121 RILs), descriptive analysis was performed under R (http://www.R-project.org).

A genetic map was constructed for the F8 JEAxCol-0 RIL population using Joinmap [32] with a LOD (logarithm of odds) score threshold of 3. Molecular markers were provided by the VNAT INRA website. Quantitative trait analysis was performed with MapQTL6 (http://www.kyazma.nl/index.php/mc.MapQTL/) using first the non-parametric Kruskal-Wallis test because in both cases the distribution of the trait was not normal. Interval mapping was also performed to determine the LOD score of the putative QTLs. The percentage of the phenotypic variation explained by the QTL corresponds to the regression value R^2^ taken at the peak LOD score of the QTL in the MapQTL3.

In order to fine map the locus associated with partial resistance to mechanically inoculated PPV in Cvi-1, a total of twenty molecular markers were developed (see Additional file 3: Table S3). Microsatellite repeat motifs were identified in *Arabidopsis* BAC sequences using Sputnik software (http://www.appliedbioinformatics.com.au/projects/ssrPrimer/cgi-bin/index). Simple sequence length polymorphism (SSLP) and Single Nucleotide Polymorphism (SNP) were retrieved from the Monsanto *Arabidopsis* Polymorphism and L*er* sequence collections (http://www.arabidopsis.org/browse/Cereon/index.jsp) using the name of the BAC clones as a search tag. Insertion Site-Based Polymorphism (ISBP) was identified by submitting the Col-0 genomic sequence to the ISBP Finder software [33]. Consequently, oligonucleotide primers complementary to the regions flanking the identified repeat motifs or polymorphic sites were designed using the program Primer version 0.5 (National Biosciences, Plymouth, Minnesota), setting an annealing temperature of 57.5 °C.

PCR fragments were amplified as described by Decroocq et al. [34]. SSR and SSLP markers were separated on 4 % agarose gels while SNP and ISBP markers were scored by High Resolution Melting curve (HRM) on a real-time PCR LightCycler. Three of the above markers (namely T18A20 SSLP1, F7A10 SSR1 and T6H22 SSR2) were tested in the F2 LCN1.12 × L*er* population. The forward primer was labeled either with FAM or with VIC and the allelic pattern of the three markers was scored among 1,736 F2 individuals in triplex on a capillary 3730 ABI sequencing machine.

To align the Cvi-1 × L*er* and JEA × Col-0 genetic maps, we selected from the above markers seven SSR and SSLP markers co-localizing with the *rpv1* locus and polymorphic between the JEA and Col-0 parental accessions (see Fig. 1). Two markers flanking the JEA × Col-0 locus were added, namely CIW1 and RCVI37. CIW1 is derived from the MSAT database (http://www.inra.fr/internet/Produits/vast/msat.php). RCVI37 is a microsatellite marker designed from the T7P1 BAC sequence, as described above (Additional file 3: Table S3). All 9 markers were screened on the full set of 250 JEA × Col-0 RILs before rerunning the quantitative analysis as described above.

### Fine-mapping of the *rpv1* locus

The fine mapping was conducted in three steps. First, a set of ten NILs, recombinant over the long arm of linkage group one, was challenged by mechanical inoculation. Then we pursued by screening 1,732 F2 plants from the LCN1.12 × L*er* cross with a set of polymorphic markers developed specifically over the *rpv1* interval determined above (Additional file 3: Table S3). Identified F2 recombinant individuals were self-pollinated and twelve to twenty-four F3 plants per F2 recombinant were challenged with PPV by mechanical inoculation. Because *rpv1* determines partial resistance to PPV infection, F3 lines having over 70 % of plants in which no PPV accumulation was observed were scored as resistant. The third step consisted in a second run of fine-mapping, this time using a LCN1.26 (susceptible) × LCN1.21 (resistant) F2 population of 840 individuals.

### Mapping of the genetic determinants in the multiparental population (MAGIC lines)

Four hundred and thirty five of the original set of 527 Multiparent Advanced Generation Inter-Cross (MAGIC) recombinant lines were tested in triplicates, following a complete random 3-block design. Included in these blocks were the 19 founders of the MAGIC lines and the PPV resistant E6 mutant. PPV accumulation in each recombinant line and founder was quantified by serological tests. Optical density values (OD) were scored as described above they were later used for quantitative analysis. The heritability among MAGIC lines was determined to best fit a random effects model [17, 16]. Using ANOVA, we determined the specific effect of ‘genotype’ and the broad-sense heritability (*h*^*2*^), which is the ratio between the genetic variance and the total phenotypic variance was calculated using the formula *h*^*2*^ *= σ*^*2*^_*g*_*/[ σ*^*2*^_*g*_ *+ (σ*^*2*^_*e*_*/n)]*, where *σ*^*2*^_*g*_ is the genetic variance, *σ*^*2*^_*e*_ is the environmental variance and *n* the number of replicates. We estimated the phenotypic mean by using an lsmean model expressed as *ls*_i_ = μ_i_ + *L* + *B* where *ls* was the lsmean value of each lines (i), *μ* represents the mean of viral accumulation for each lines (i), *L* and *B* were estimations of the lines and block effects, respectively. Procedures for statistic and QTL analyses are described elsewhere [16, 5].

### Association mapping

One hundred and forty-seven accessions previously genotyped with 216,000 SNPs [35] were mechanically inoculated following a complete random 4-block design, the experiment was repeated over two years. The broad-sense heritability (*h*^*2*^) was evaluated as described above. Quantitative data for viral accumulation and the estimation of the phenotypic mean (lsmean model) were generated as described for the MAGIC lines. We chose to combine the two experiments by considering the 8 blocks as a single data set in the lsmean model. To resolve the phenotypic scoring into binary data, thus avoiding intermediate scores, we decided to rate accessions as susceptible (an assigned value of 1) when the lsmean value was at least three times the lsmean value of the PPV resistant E6 negative control [13].

Genotypic data (mostly Single Nucleotide Polymorphism) were assigned as described in Atwell et al. [35] and are publically available at the AtPolyDB database (https://easygwas.tuebingen.mpg.de/data/public/dataset/view/1/).

Fisher’s exact tests were implemented on binary data to test for the association between genotypes and phenotypes. For quantitative data we used a Wilcoxon rank sum tests in addition a regression analysis was implemented using the Plink software [36] (http://pngu.mgh.harvard.edu/purcell/plink/). All those analyses were expected to have false positives due to population structure. Thus, we also used the EMMA method [37] based on a mixed model that accounts for effects due to population structure.

### *cPGK2* Virus-Induced Gene Silencing (VIGS) in *Benthamiana*

*The Tobacco rattle virus* (TRV) based VIGS system was used to knock down the expression of host genes. Construction of the pTRV1, pTRV2/PGK-5, pTRV2/PGK-3 and pTRV2/PDS plasmids is described in Ouibrahim et al. [9]. Two plasmids pTRV2/PGK-5 and pTRV2/PGK-3 containing fragments corresponding to 5’ and 3’ ends of tobacco chloroplast PGK, were used to knock down the *Arabidopsis* AT1G56190 chloroplast PGK. Plasmid pTRV2/PDS containing a phytoene desaturase was used as a positive control of silencing [12].

*Nicotiana benthamiana* was grown in BL-3 containment greenhouse under the controlled conditions of temperature (20 °C) and relative humidity (60 %). *Agrabacterium tumefaciens* cultures at OD_600_ = 1 containing pTRV1 or pTRV2 derivative plasmids were mixed in 1:1 ratio and infiltrated with a syringe onto two leaves of each plant. Twelve days after infiltration, newly formed leaves were mechanically inoculated with the pICPPVnkGFP clone. Six days post inoculation (dpi), newly-formed, non-inoculated leaves were sampled.

Total RNA was extracted from *N. Benthamiana* using the SV Total RNA Isolation System® from Promega Biosciences, LLC. First strand cDNA was synthesized from total RNA using Superscript II® reverse transcriptase from Invitrogen. Quantitative RT-PCR (Q-RT-PCR) was performed on a Light Cycler 480 II machine (Roche Diagnostics) by using LightCycler® 480 SYBR Green I master and one tenth of the newly synthesized cDNAs. The chloroplast-specific PGK was amplified and detected by a forward primer (5′-GCCTTCTGTTGCAGGTTTCC-3′) and a reverse primer (5′-ATTCCTCCACCCAAAAGCAA-3′). PCR was performed using the following cycling conditions: 95 °C for 5 min, and 40 cycles of 95 °C for 30 s, 60 °C for 30 s and 72 °C for 45 s followed by a melting curve ramp from 72 °C to 95 °C for 10 s. Q-RT-PCR experiment was conducted on four to six biological replicates for each sample. For comparison of the data among experimental samples, the real-time PCR results were normalized using the levels of *N. benthamiana* elongation factor 1 (NbEF1) mRNA using a forward primer (5′-GATTGGTGGTATTGGAACTGTC-3′) and a reverse primer (5′-AGCTTCGTGGTGCATCTC-3′) [38].

### *cPGK2* expression analysis in *Arabidopsis*

Rosette leaves of L*er*, Col-0 and Cvi-1 were harvested just before inoculation. RNA was isolated by using the Macherey Nucleospin Total RNA isolation kit. After RNA extraction, RNA was diluted to 50 to 100 ng/μL with DEPC treated water. Reverse transcription was done by using 0.5 μg of total RNA and Revertaid/Ribolock reverse transcriptase kit (Fermentas). Q-RT-PCR was performed as described above. The *Arabidopsis cPGK2* primers used for Q-RT-PCR analysis were: forward CCTCCTTTGGACACATTCCC and reverse ATCTCCAACACTCTTCTTCGC. PCR was performed using the following cycling conditions: 95 °C for 5 min, and 40 cycles of 95 °C for 30 s, 59 °C for 30 s and 72 °C for 45 s followed by a melting curve ramp from 72 °C to 95 °C for 10 s. Two independent Q-RT-PCR experiments were conducted, testing three plants per accession in each experiment.

Relative expression was calculated using the Efficiency method (Roche Diagnostics) in comparison with the At2g36060 endogenous control [19]. Fold change in *cPGK2* expression was determined relative to the reference gene.

## Additional files

Additional file 1: Table S1. Arabidopsis accessions genotyped with the full 250 K SNPs data set and challenged with PPV by mechanical inoculation. The experiment was repeated twice and in each experiment, all accessions were tested in four replicates, following a complete random 4-block procedure. Additional file 2: Table S2. Most informative SNPs in the 500 top markers when testing genome wide association with restricted PPV systemic infection in *Arabidopsis* accessions. (A) Results of the mean binary data, after Fisher analysis. (B) Results of the quantitative data after Wilcoxon rank sum test. (C) Results of the quantitative data after PLINK method. (D) Results of the quantitative data after EMMA method. *: SNPs belonging to the *rpv1* interval (see Table 1). φ: SNPs belonging to the *sha3* interval [55]. Additional file 3: Table S3. Primers use to fine map *rpv1* in LCN near isogenic lines and JEA x Col-0 RIL population *: Base pair. Additional file 4: Figure S1. Relative expression of the *cPGK2* (At1g56190) gene in *Arabidopsis* accessions. The relative expression of the *Arabidopsis cPGK2* gene (At1g56190) in rosette leaves, before PPV infection, was calculated in comparison with the C34260 reference gene corresponding to At2g36060. The experiment was repeated twice, depicted in light and dark grey colors.

## Abbreviations

Col-0: Columbia-0
cPGK: chloroplastic phosphoglycerate kinase
Cvi-0 and −1: Cape Verde Island-0 and −1
GWAS: Genome Wide Association Study
ISBP: Insertion Site-Based Polymorphism
L*er*: Landsberg *erecta*
LOD: logarithm of odds
MAGIC: Multiparent Advanced Generation Inter-Cross
NIL: Near Isogenic Line
PPV: Plum Pox Virus
Q-RT-PCR: Quantitative Reverse Transcribed Polymerase Chain Reaction
QTL: Quantitative trait locus
RIL: Recombinant Inbred Line
SNP: Single Nucleotide Polymorphism
SSLP: Simple sequence length polymorphism
VIGS: Virus-induced gene silencing
