# Supplementary material for: Allelic variation at the rpv1 locus controls partial resistance to Plum pox virus infection in Arabidopsis thaliana
Source: BMC Plant Biol. 2015 Jun 25;15:159. doi: 10.1186/s12870-015-0559-5 (PMC4479089; doi:10.1186/s12870-015-0559-5)
Supplement: Additional file 1: Table S1. — Arabidopsis accessions genotyped with the full 250 K SNPs data set and challenged with PPV by mechanical inoculation. The experiment was repeated twice and in each experiment, all accessions were tested in four replicates, following a complete random 4-block procedure. [file 12870_2015_559_MOESM1_ESM.docx]

**Table S1. Arabidopsis accessions genotyped with the full 250K SNPs data set and challenged with PPV by mechanical inoculation**

| **ID NASC** | **Accession** | **ID NASC** | **Accession** | **ID NASC** | **Accession** | **ID NASC** | **Accession** |
| --- | --- | --- | --- | --- | --- | --- | --- |
| N923 | H55 | N6002 | PHW-2 | N22580 | Var2-1 | N22626 | An-1 |
| N926 | Petergof | N6003 | Köln | N22586 | Ull2-5 | N22627 | Van-0 |
| N927 | Rubezhnoe-1 | N6033 | Lisse | N22587 | Ull2-3 | N22628 | Br-0 |
| N949 | Ang-0 | N6041 | Kelsterbach 4 | N22588 | Zdr-1 | N22629 | Est-1 |
| N971 | Bla-1 | N6187 | Seattle-0 | N22589 | Zdr-6 | N22630 | Ag-0 |
| N997 | Bs-1 | N6600 | Aa-0 | N22590 | Bor-1 | N22631 | Gy-0 |
| N1007 | Bu-0 | N6607 | Ba-1 | N22591 | Bor-4 | N22632 | Ra-0 |
| N1031 | Blh-1 | N6658 | Ca-0 | N22592 | Pu2-7 | N22633 | Bay-0 |
| N1065 | Can-0 | N6676 | Da-0 | N22593 | Pu2-23 | N22634 | Ga-0 |
| N1067 | Cen-0 | N6697 | Ep-0 | N22594 | Lp2-2 | N22635 | Mrk-0 |
| N1137 | En-1 | N6720 | Gie-0 | N22595 | Lp2-6 | N22636 | Mz-0 |
| N1185 | Gd-1 | N6733 | Ha-0 | N22596 | HR-5 | N22637 | Wt-5 |
| N1187 | Ge-0 | N6751 | Kas-2 | N22597 | HR-10 | N22639 | Ct-1 |
| N1199 | Gr-1 | N6778 | Li-7 | N22598 | NFA-8 | N22640 | Mr-0 |
| N1227 | Hi-0 | N6792 | Mh-0 | N22599 | NFA-10 | N22641 | Tsu-1 |
| N1237 | Hs-0 | N6822 | Or-0 | N22600 | Sq-1 | N22642 | Mt-0 |
| N1239 | In-0 | N6842 | Pog-0 | N22601 | Sq-8 | N22643 | Nok-3 |
| N1259 | Jm-0 | N6864 | Ste-0 | N22602 | CIBC-5 | N22644 | Wa-1 |
| N1307 | Lc-0 | N6903 | Zu-1 | N22603 | CIBC-17 | N22645 | Fei-0 |
| N1337 | Lip-0 | N6921 | Cnt-1 | N22604 | Tamm-2 | N22646 | Se-0 |
| N1345 | Lm-2 | N8067 | Buckhorn Pass | N22606 | Kz-1 | N22647 | Ts-1 |
| N1353 | Lu-1 | N8143 | WAR | N22607 | Kz-9 | N22648 | Ts-5 |
| N1379 | Mir-0 | N22342 | BG2 | N22608 | Got-7 | N22649 | Pro-0 |
| N1385 | Na-1 | N22449 | Pu2-8 (3-8) | N22609 | Got-22 | N22651 | Kondara |
| N1431 | Ost-0 | N22491 | N13 | N22610 | Ren-1 | N22652 | Sakhdara |
| N1439 | Pa-1 | N22564 | RRS-7 | N22611 | Ren-11 | N22653 | Sorbo |
| N1445 | Per-1 | N22565 | RRS-10 | N22612 | Uod-1 | N22654 | Kin-0 |
| N1482 | Rd-0 | N22566 | Knox-10 | N22613 | Uod-7 | N22656 | Bur-0 |
| N1485 | Rak-2 | N22567 | Knox-18 | N22614 | Cvi-0 | N22658 | Oy-0 |
| N1494 | Rsch-4 | N22568 | Rmx-A02 | N22615 | Lz-0 | N22659 | Ws-2 |
| N1507 | Sap-0 | N22569 | Rmx-A180 | N22616 | Ei-2 |  |  |
| N1515 | Sav-0 | N22570 | Pna-17 | N22617 | Gu-0 |  |  |
| N1535 | St-0 | N22571 | Pna-10 | N22618 | Ler-1 |  |  |
| N1539 | Stw-0 | N22573 | Eden-2 | N22619 | Nd-1 |  |  |
| N1549 | Ta-0 | N22574 | Löv-1 | N22620 | C24 |  |  |
| N1567 | Tu-0 | N22575 | Löv-5 | N22622 | Wei-0 |  |  |
| N1656 | Alc-0 | N22576 | Fäb-2 | N22623 | Ws-0 |  |  |
| N3081 | No-0 | N22577 | Fäb-4 | N22624 | Yo-0 |  |  |
| N3180 | Co | N22579 | Bil-7 | N22625 | Col-0 |  |  |

The experiment was repeated twice and in each experiment, all accessions were tested in four replicates, following a complete random 4-block procedure.
