# Supplementary material for: Allelic variation at the rpv1 locus controls partial resistance to Plum pox virus infection in Arabidopsis thaliana
Source: BMC Plant Biol. 2015 Jun 25;15:159. doi: 10.1186/s12870-015-0559-5 (PMC4479089; doi:10.1186/s12870-015-0559-5)
Supplement: Additional file 3: Table S3. — Primers use to fine map rpv1 in LCN near isogenic lines and JEA x Col-0 RIL population *: Base pair. [file 12870_2015_559_MOESM3_ESM.docx]

**Table S3. Primers use to fine map *rpv1* in LCN near isogenic lines and JEA x Col-0 RIL population**

| **Name** | **Position in bp*** | **Type** | **Forward** | **Reverse** | **Tm** |
| --- | --- | --- | --- | --- | --- |
| RPVm2 / CIW1 | 18367546 | SSR | ACATTTTCTCAATCCTTACTC | GAGAGCTTCTTTATTTGTGAT | 55°C |
| F6D8-SSLP1 | 19624423 | SSLP | TTTCGGGTGATTAGTACGGA | ATTTGACTCCGTCTTCCAGA | 55°C |
| F12M16-SSR1 | 19840749 | SSR | AAAAACCTTCCGTGAGAGTGC | GAGAAGACGATTATGGTGATGAT | 55°C |
| F12M16-SSR2 | 19860943 | SSR | TCACGGCCCATTAGTGTTCT | TTTCTCAAACGCGAAAGCC | 55°C |
| T18A20-SSLP1 | 20075679 | SSLP | TTCGTAATGAAATGAACTGAGC | CCATTGACCAAGTTATCATTTATG | 55°C |
| F20D21-SSR2 | 20255382 | SSR | TCCTTAACCGATGTAATGTCCA | TCTTCTGGTGCTCTTGGTACG | 55°C |
| F14C21-SSR2 | 20510667 | SSR | AGAGTTGACAAGCAAAGGGA | CAAAACGTCGATCATCTTCA | 55°C |
| NGA-128 | 20633249 | SSR | GGTCTGTTGATGTCGTAAGTCG | ATCTTGAAACCTTTAGGGAGGG | 55°C |
| F20N2-SSR1 | 20777881 | SSR | CGAGTCCAGAGCACGAAC | CTTTTCCACTGTGAACGAAGA | 55°C |
| NGA-280 | 20877363 | SSR | GGCTCCATAAAAAGTGCA | CTGATCTCACGGACAATAGTGC | 55°C |
| F14J16-SSR2 | 20933254 | SSR | TTACCTTGATTGAGAGTTCGC | CTCCTTCATATCTTCTTTTCCACT | 55°C |
| T6H22-SSR2 | 20966441 | SSR | ACATGAATGGGTATTATC | CAAGAACAATGAGAGCGA | 55°C |
| T6H22-SNP1 | 20971975 | SNP | TATCAGCCACCAAAACTGAAGCC | CAGGTTATCCCCACAGCCAGAA | 58°C |
| T6H22-SSR1 | 20983250 | SSR | GTTTCATCTCTCCACGTTCC | TTGGGTTTTCCATAAACTCTCA | 55°C |
| F14G9-SNP3 | 21094499 | SNP | GGAAGACATAGAGTTCTGGGTC | GCCCAAGGTTTGTTGGTT | 58°C |
| ISBP-16 | 21232619 | ISBP | CCGACAACTTCTAAGCTCCG | GCTTCACACTCCCTAGCCAA | 60°C |
| F25P12-SNP2 | 21253016 | SNP | CTCAACTTCACAAGTCAACACTG | GGCACATATCAATCTTAGTAAGAT | 53°C |
| F25P12-SNP1 | 21318292 | SNP | AACTGTCGGGTACACGCTTC | TATCCGCAAAGTTTGGAACC | 53°C |
| T8L23-SSR | 21324252 | SSR | TGTAGCATATTCCTCTGACTGG | GGAATCAAACCAACGACCTA | 55°C |
| T8L23-SNP2 | 21379240 | SNP | TAAGCTGCATGAATCTTTCCACTG | GGATCCTCTGTTCTTGTTCTG | 53°C |
| F12K22-SSR1 | 21428414 | SSR | CAAGCATGCATATATTATTGTTAA | AATTTGTCTAGTTTTCAGGGATGT | 55°C |
| T15M6-SSR1 | 21541832 | SSR | ATCAAATTCATCACAGAATCCTTT | GAGAAGGTAACTAATTCCAAACA | 55°C |
| F16M22-SSR2 | 21605239 | SSR | GCAAGGTTTTGGAAGTGTGA | CGATGGTCGATGGGTTTAGG | 55°C |
| RCVI-23 | 22015943 | SSLP | CGTTAAATCCAAAAGCTGTGT | AACAAGAAGCACCATTGACAG | 55°C |
| RCVI-1 | 22255648 | SSR | ATTTAAATGCATGGAGAATAGAAG | AGGAAGCTAGTTCTAACGACATCT | 55°C |
| RCVI-37 | 22472690 | SSR | TTCCGATCAAAGGAACGC | TTCTGACTTAGATAGTGCGTATGG | 55°C |

***Base pair**
