# Supplementary figures and images for: Allelic variation at the rpv1 locus controls partial resistance to Plum pox virus infection in Arabidopsis thaliana
Source: BMC Plant Biol. 2015 Jun 25;15:159. doi: 10.1186/s12870-015-0559-5 (PMC4479089; doi:10.1186/s12870-015-0559-5)

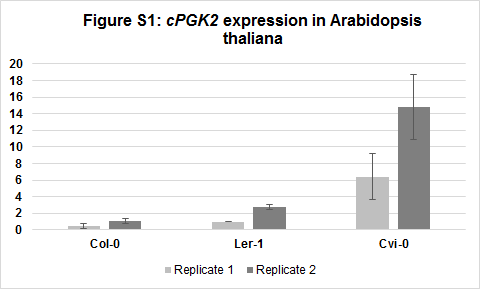

Supplement: Additional file 4: Figure S1. — Relative expression of the cPGK2 (At1g56190) gene in Arabidopsis accessions. The relative expression of the Arabidopsis cPGK2 gene (At1g56190) in rosette leaves, before PPV infection, was calculated in comparison with the C34260 reference gene corresponding to At2g36060. The experiment was repeated twice, depicted in light and dark grey colors. [file 12870_2015_559_MOESM4_ESM.tif]
